# Supplementary material for: Cissus quadrangularis (Hadjod) Inhibits RANKL-Induced Osteoclastogenesis and Augments Bone Health in an Estrogen-Deficient Preclinical Model of Osteoporosis Via Modulating the Host Osteoimmune System
Source: Cells. 2023 Jan 4;12(2):216. doi: 10.3390/cells12020216 (PMC9857034; doi:10.3390/cells12020216)
Supplement: Supplementary file 1 [file cells-12-00216-s001.zip › cells-2060760-supplementary.pdf]

cells

MDPI

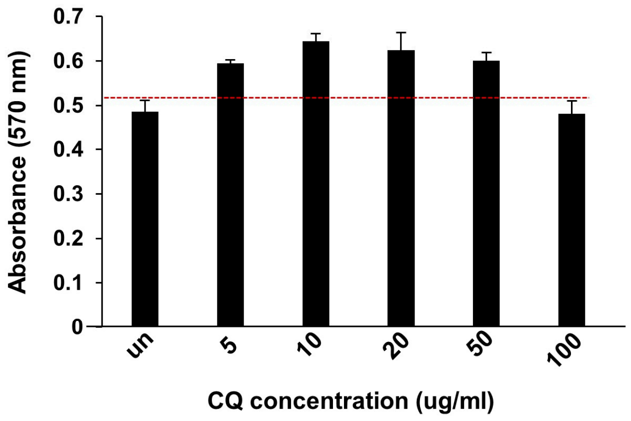

Figure S1: Cell cytotoxicity assay. Bone marrow cells (BMCs) were treated with different concentrations of CQ for 48 h and MTT assay was performed for evaluating the cytotoxic effects of CQ.

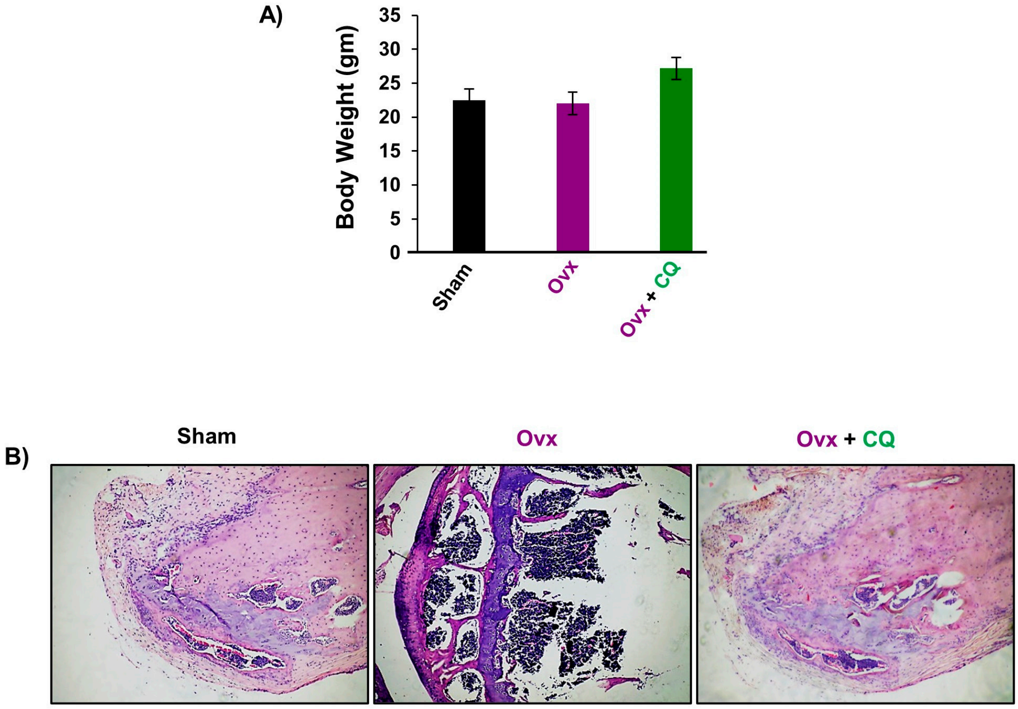

Figure S2: Histological features of bone specimens: A) Body weight. **B)** Representative H & E staining of femoral bone sections from each group at 10 X Magnification.

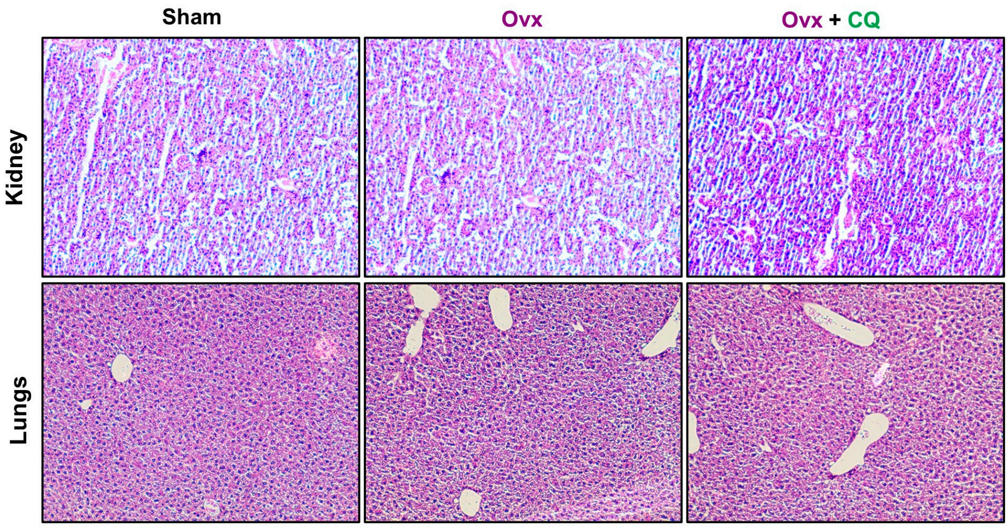

Figure S3: Histopathological analysis of kidney and lung tissues: H & E staining of kidney and lung tissue sections in mice of all three groups.

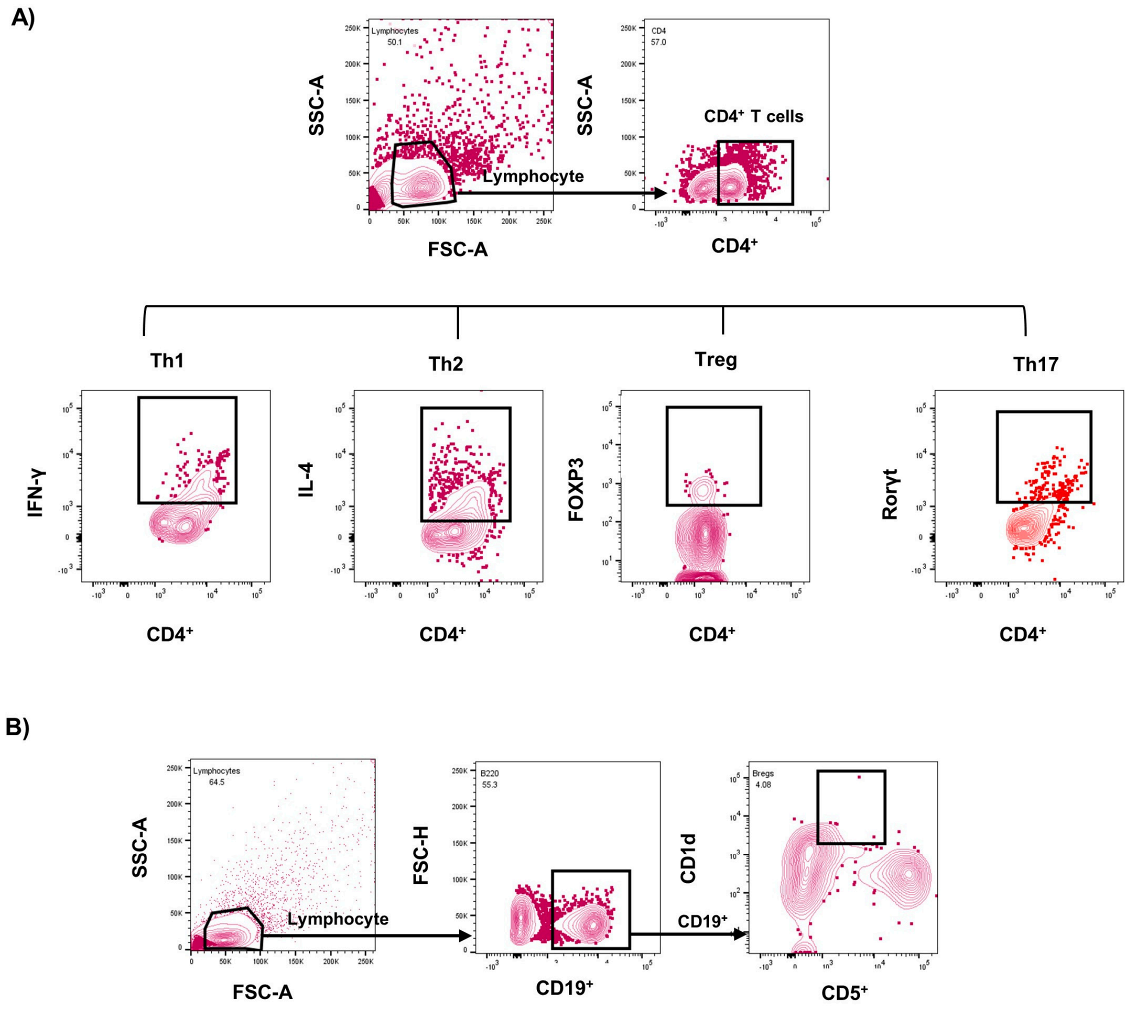

Figure S4: Gating strategy followed for flow data analysis: A) Gating strategy for Th1, Th2, Th17 and Tregs and **B)** Gating strategy for Bregs.
